# Predictors of emotional intelligence among family caregivers of cancer patients: A cross‐sectional study

**DOI:** 10.1002/cnr2.1943

**Published:** 2023-11-22

**Authors:** Shakiba Moosivand, Omid Nazari, Ali Shahverdi, Mohammad Gholami, Rasool Mohammadi, Sajad Yarahmadi

**Affiliations:** ^1^ Student Research Committee, School of Nursing and Midwifery Lorestan University of Medical Sciences Khorramabad Iran; ^2^ Social Determinants of Health Research Center, School of Nursing and Midwifery Lorestan University of Medical Sciences Khorramabad Iran; ^3^ Nutritional Health Research Center, School of Health and Nutrition Lorestan University of Medical Sciences Khorramabad Iran; ^4^ Student Research Committee Semnan University of Medical Sciences Semnan Iran

**Keywords:** cancer patients, coping, emotional intelligence, family caregiver, pain control, spiritual intelligence

## Abstract

**Background:**

Family caregivers of cancer patients must have strong emotional intelligence skills essential for understanding one's and others' feelings and learning how to cope.

**Objectives:**

The present study aims to determine the predictive factors of emotional intelligence and its relationship with spiritual intelligence, coping, and knowledge and experience about pain among family caregivers of cancer patients in Iran.

**Methods:**

A cross‐sectional, descriptive study was conducted in 2020–2021. Two hundred twenty‐six family caregivers of cancer patients participated in this study. The data collection tools were Wong and Law's emotional intelligence, King's spiritual intelligence, Brief Cope, and the family pain questionnaire. Following determining the variables' correlation, linear regression was carried out.

**Results:**

Emotional intelligence had a significant correlation with age (*r* = 0.20, *p* = .003), academic degree (*r* = 0.15, *p* = .032), duration of care (*r* = 0.15, *p* = .032), and spiritual intelligence (*r* = 0.30, *p* < .001). The regression model accounted for 12.4% of the variance in emotional intelligence; age (*β* = 0.16, *p* < .021) and spiritual intelligence (*β* = 0.26, *p* < .001) were significant explanatory variables.

**Conclusions:**

Emotional intelligence is correlated with age, academic degree, duration of care, and spiritual intelligence, but only age and spiritual intelligence were found to be predictive factors for emotional intelligence in the family caregivers of cancer patients.

## 1 INTRODUCTION

Cancer is the second most significant cause of death worldwide.^1^ Cancer patients experience multiple symptoms during their illness and treatment.^2^ In addition, advanced‐stage patients depend more on others and have limited functioning due to their illness. As a result, family caregivers play an essential role and must be acknowledged as part of the care process.^3^ Various multidimensional factors, such as disease trends, hospital policies, financial issues, and accessibility and communication of healthcare services, can impact patients and their family caregivers; therefore, they require support.^4^ However, caring for a family member with cancer appears to be detrimental to family caregivers' emotional and physical health.^5^ Family caregivers frequently serve as the patient's primary source of social and emotional support, significantly impacting how effectively patients deal with their disease's and treatment's effects.^6^ Based on emotional and social intelligence, the ability to communicate determines how well family members can get along and adapt to new living conditions.^7^

Emotional intelligence has been identified as a crucial personal resource in research. It is described as having the ability to process emotional information, such as recognizing, creating, and controlling emotions, data on how to express emotions in oneself and others, and information on how to manage emotions for emotional development. Finding strategies to manage and adapt to one's own and other people's feelings is a crucial component of emotional intelligence.^8^ Features of emotional intelligence play a critical role in overcoming stressful situations and carrying out good interpersonal interactions with family patients. The ability to perceive and comprehend one's emotional states and other people's emotional states forms the basis for delivering effective and adaptable emotional and behavioral responses in a family‐friendly atmosphere.^7^ Few studies have been conducted on the emotional intelligence of families of cancer patients, and it is necessary to identify factors related to or promoting their emotional intelligence.

For caregivers, dealing with caregiving problems often involves their spirituality. Spirituality is the fundamental element of palliative care that enhances the quality of life of family members of patients with life‐threatening diseases.^9^ For family caregivers of cancer patients, spirituality may be a source of strength and a useful resource.^10^ In Seo La et al. study, among caregivers, spirituality is associated with a better sense of meaning and purpose and a lower risk of depression.^11^ Emons defines the emergence of spiritual intelligence as the use of spiritual resources and capacities in real‐world contexts and situations. In other words, people employ spiritual intelligence when they desire to harness spiritual resources and talents to make crucial decisions, consider existential issues, or attempt to address everyday problems.^12^ A study showed a correlation between emotional and spiritual intelligence.^13^

Actions taken to resolve or lessen a stressful circumstance are called coping. People utilize adaptive (approach coping) and maladaptive (avoidant coping) coping mechanisms to deal with threats and maintain mental stability.^14^ Several studies have been conducted on coping with family caregivers of cancer patients. According to Long et al., cancer caregivers can lessen their caring load, ease psychological stress, and enhance their quality of life when they have a solid social support network and use appropriate coping strategies.^15^ Additionally, Akpan‐Idiok et al. revealed that carers' coping mechanisms were related by age, sex, educational status, functional capacity, length of care, and motivation to continue providing care.^1^ Ghafoor et al. study revealed a relationship between low emotional intelligence and maladaptive coping strategies.^16^ Furthermore, a later study (2020) confirms the correlation between emotional intelligence and coping in other societies.^17^

Pain is one of the most frequent symptoms that cancer patients encounter, significantly affecting their work, activities, motivation, mood, and overall quality of life.^18^, ^19^, ^20^ When left uncontrolled, it deprives them of comfort. Moreover, with the shift of healthcare services from hospitals to the community, many cancer patients receive treatment for prolonged periods with the aid of family caregivers. The patients' suffering is greatly influenced by their individual traits, understanding of cancer pain, and the attitudes of their family caregivers.^21^

The knowledge, attitude/belief, and practice model contends that patients' perceptions of pain impact their beliefs and then impact how they manage their pain, which will impact how well they manage their pain. It should also be noted that family caregivers play a vital role in providing comprehensive cancer pain assessments, communicating with medical staff, administering pain medications, and managing associated side effects. As a result, it is assumed that family members' knowledge of pain will likewise impact the pain outcomes of patients.^21^ Providing sufficient pain management frequently falls on unpaid caregivers who feel underqualified for the high expertise and knowledge required.^22^ An earlier study found that patients with cancer who perceived their pain differently from their family caregivers had more serious concerns about reporting pain and taking painkillers. They were also more likely to experience worse pain.^23^ Since emotional intelligence is defined as the capacity to express emotions in oneself and to others,^8^ according to the researchers' hypothesis, there may be a relationship between the experience and knowledge of pain control of the family of cancer patients and emotional intelligence. During the searches, no studies show the relationship between these two variables. Numerous studies and literature have examined the impact of demographic traits on individuals' emotional intelligence, but their findings are inconsistent.^24^ For instance, while one study identified age as a predictor of emotional intelligence, another found no correlation.^25^

According to the available evidence and our hypotheses, the current study aimed to identify the factors that predict emotional intelligence in family caregivers of cancer patients and their associations with demographic data, spiritual intelligence, coping, and knowledge and experience of pain control.

## 2 METHODS

### 2.1 Design

A cross‐sectional descriptive study was conducted from December 2020 to March 2021, employing this design to offer a comprehensive overview of the research topic's current status and explore the interplay of various variables within the selected sample.

### 2.2 Participants

The participants of this research were families of cancer patients referred to the oncology department of Shahid Rahimi Hospital, Khorramabad, Iran.

The inclusion criteria for this study were family caregivers of cancer patients who had been diagnosed for at least 1 month, were between the ages of 18 and 65, had completed at least a high‐school education, and were willing to participate. The exclusion criteria needed to respond to at least one of the four questionnaires and a lack of interest to continue the study were considered exclusion criteria.

### 2.3 Sampling

Convenience sampling was chosen as the sampling method for this study due to its practicality and accessibility in obtaining data from the available population within the given time and resource constraints, including family caregivers who were admitted into the hospital for inpatient care or attended care services, including the outpatient department (OPD).

### 2.4 Sample size

The sample size is based on the previous study,^26^ taking into account the standard deviation of the matching score (*σ* = 11.51) and *α* = 0.05, *d* = 1.5, Z1‐*α*/2 = 1.96, the number of 226 people was calculated using the following formula and taking 10% of potential attrition in the sample into account. Out of 226 questionnaires, 211 questionnaires (94%) were completed.

$$n=\frac{\sigma^{2}Z_{1-\frac{\alpha }{2}}^{2}}{d^{2}}$$

### 2.5 Instruments

Five questionnaires were used as data‐gathering instruments. The King's spiritual intelligence, brief cope scale, and Family Pain Questionnaire were all used with permission from the instruments' developers. The demographic data questionnaire included eight questions: age, gender, marital status, academic degree, relationship with the patient, employment status, cancer grade, and duration of care.

### 2.6 Emotional Intelligence

Wong and Law's emotional intelligence questionnaire (2002) consists of 16 items and includes four dimensions: evaluation and expression (1–4); emotional understanding and analysis (5–8); use of emotions to facilitate thought (9–12); and emotional regulation (13–16). A 5‐point Likert scale was assigned scores (1 = completely opposed, 5 = completely agree). High scores indicate higher emotional intelligence. This questionnaire has a 0.84 Cronbach's *α* coefficient.^27^

### 2.7 Spiritual Intelligence

King's self‐report questionnaire for measuring spiritual intelligence includes four dimensions: existential critical thinking (1, 3, 5, 9, 13, 17, and 21), personal meaning production (7, 11, 15, 19, and 23), transcendental awareness (2, 6, 10, 14, 18, 20, and 22), and development of consciousness (4, 8, 12, 16, and 24). The questionnaire comprised 24 items on a 5‐point Likert scale (1 = completely false, 5 = completely correct). A higher score implies a higher level of spiritual intelligence. By measuring Cronbach's alpha coefficient, the reliability of this questionnaire was 0.87.^28^

### 2.8 Brief‐COPE scale

The 28‐item Brief‐COPE scale was developed by Buchanan to evaluate various coping strategies. Scores are shown for the two main coping styles: Denial, substance use, venting, behavioral disengagement, self‐distraction, and self‐blame are the subscales that define avoidant coping (1, 3, 4, 6, 8, 9, 11, 13, 16, 21, and 26). Active coping, positive reframing, planning, acceptance, seeking emotional support, and seeking informational help are the subscales that define approach coping (2, 5, 7, 10, 12, 14, 15, 17, 19, 20, 23, 24, and 25). Humor (18 and 28) and religion (22 and 27) are neither approaches nor avoidance coping. Scoring was done using a 4‐point Likert scale (1 = I have not been doing this at all, 4 = I have been doing this a lot). A higher rating will indicate better‐coping skills.^29^ The researchers translated this tool, assessed its reliability and validity, and localized it to Iranian society for the first time. The validity of each item was good. Cronbach's *α* coefficient of this questionnaire is 0.77.

### 2.9 Knowledge and experience with pain

Ferrell et al. developed the 16‐item Family Pain Questionnaire as a self‐report tool to evaluate a family caregiver's knowledge (1–9) and experience (10–16) in managing chronic cancer pain. A 10‐point Likert scale was assigned scores (0 = the most positive outcome and 10 = the most negative outcome). High scores show a family caregiver's knowledge and experience managing chronic cancer pain.^30^ The researchers translated this tool, assessed its reliability and validity, and localized it to Iranian society for the first time. Based on the overall content validity results, 15 items remained after one was removed (item 9). The questionnaire's Cronbach's *α* coefficient is 0.60.

### 2.10 Adaptation of Brief‐COPE and Family Pain Questionnaire

The forward‐backward method was used to translate the original English versions into Persian. Ten family caregivers of cancer patients completed the scales during the face validity phase. To assess the content validity, both qualitative and quantitative methods were employed. Ten faculty members with expertise in instrument development, patient care, and psychology were asked to evaluate and comment on the questions' wording, item allocation, and scaling during the qualitative stage. The Content Validity Ratio (CVR) and Content Validity Index (CVI) were computed. On a three‐point scale, 10 experts were asked to rank each item for reporting CVR as necessary, useful but not necessary, or unnecessary. According to Lawshe's table, the items with a CVR of 0.62 and higher were conserved.^31^ The same expert panel's scoring was used to calculate the CVI for each item's coefficient. A CVI value of 0.79 or higher was deemed ideal in the final versions without re‐reviewing.^32^ Cronbach's *α* coefficient was used to evaluate the method's reliability. Fifty family members of cancer patients completed the questionnaires.

### 2.11 Procedure

This study was conducted after obtaining consent from the Lorestan University of Medical Sciences. The researchers, who were three trained undergraduate nursing students, visited the site and explained the study's objectives to the participants before distributing the questionnaire. The data were collected through self‐reporting using both paper and electronic questionnaires. Participants who had internet access completed the questionnaire electronically by following the link provided by the researcher, while others completed a paper questionnaire. The study participants were also given access to the researcher's email address and phone number.

### 2.12 Statistical analyses

The general characteristics and responses of the participants regarding emotional intelligence, spiritual intelligence, coping, and knowledge and experience of pain were summarized using descriptive statistics. Mean comparison techniques were used to assess the variations in emotional intelligence based on demographic characteristics. The study hypotheses were evaluated in the inferential section using the intragroup correlation coefficient, Pearson (normally distributed variables and linear relationships), Spearman (ordinal scale or non‐normally distributed variables) correlation tests, and linear regression analysis. Before conducting the regression analysis, we verified that our data satisfied the fundamental regression assumptions, such as the homogeneity of variance and multicollinearity. We utilized imputation for the missing data (mean substitution). The alpha error level was set to a maximum of 0.05. The data analysis was conducted using IBM SPSS Statistics 22.0. According to the Kolmogorov–Smirnov and Shapiro–Wilk tests, emotional intelligence, spiritual intelligence, coping, and knowledge and experience of pain variables had a normal distribution (*p* > .05).

## 3 RESULTS

Due to incomplete questionnaires, 15 participants were excluded from the study. In our study, a total of 5% of the data was missing, indicating a relatively small proportion of missing observations in our dataset. We conducted rigorous statistical analyses, including Little's MCAR test, which confirmed that the messiness mechanism is missing at random (MAR).

### 3.1 Descriptive Statistics

In this study, 211 family caregivers of cancer patients with a mean age of 33.3 (9.55) years (118 men and 93 women) participated. The mean duration of care was 13.7 (13.42) months. In Table 1, further demographic information is provided.

**TABLE 1:** Description of demographic variables and emotional intelligence.

Variables	*N* (%)	Emotional intelligence mean (SD)
*Age (years)*		
18–30	89 (42.20)	59.62 (8.39)
31–45	106 (50.20)	62.07 (7.70)
≥45	16 (7.60)	66.25 (9.01)
*Gender*		
Male	118 (55.90)	61.32 (8.57)
Female	93 (44.10)	61.44 (7.90)
*Marital status*		
Single	70 (33.20)	60.73 (8.67)
Married	137 (64.90)	62.38 (7.33)
Divorced	3 (1.40)	61.63 (7.25)
Widow/widower	1 (0.50)	75.15 (−)
*Academic degree*		
Diploma	98 (46.40)	62.07 (6.47)
BS	94 (44.50)	59.41 (8.50)
MS and upper	19 (9.00)	63.08 (8.01)
*Relation*		
Father	6 (2.80)	61.47 (13.06)
Mother	20 (9.50)	57.60 (9.10)
Brother	9 (4.30)	63.32 (9.16)
Sister	18 (8.50)	62.25 (8.29)
Spouse	42 (19.90)	61.32 (7.15)
Child	116 (55.00)	61.69 (8.22)
*Employment status*		
Unemployed	57 (27.00)	61.39 (7.76)
Housewife	64 (30.30)	60.02 (8.98)
University student	15 (7.10)	59.55 (7.81)
Employed	63 (29.90)	62.80 (7.88)
Retired	12 (5.70)	62.97 (8.97)
*The patient's cancer grade*		
Grade 1	37 (17.50)	60.61 (7.70)
Grade 2	54 (25.60)	61.79 (8.06)
Grade 3	37 (17.50)	59.92 (7.66)
I do not know	83 (39.30)	62.12 (8.90)
*Duration of care (months)*		
1–12	142 (67.30)	59.95 (8.14)
13–36	50 (23.70)	59.89 (8.56)
≥37	19 (9.00)	62.09 (8.13)

The results showed that in family caregivers of cancer patients, the mean (S.D.) emotional intelligence was 61.37 (8.26), coping was 74.31 (9.28), spiritual intelligence was 84.11 (14.58), and knowledge and experience in pain control was 81.44 (18.30) (Table 2).

**TABLE 2:** Describing the level of the main variables and their dimensions among the participants.

Variable (min–max)	Dimensions	Dimensions mean (SD)	Total mean (SD)
Emotional intelligence (16–80)	Evaluation and expression	15.70 (2.51)	61.37 (8.26)
	Emotional, Understanding, and Analysis	15.28 (2.59)	
	Use of emotions to facilitate thought	16.17 (2.52)	
	Emotional regulation	14.05 (3.29)	
Coping (28–112)	Avoidant	22.62 (4.84)	74.31 (9.28)
	Approach	41.88 (5.87)	
	Humor	3.43 (1.74)	
	Religion	6.35 (1.64)	
Spiritual intelligence (24–120)	Existential critical thinking	25.27 (5.17)	84.11 (14.58)
	Personal meaning production	15.07 (2.93)	
	Transcendental awareness	23.73 (4.55)	
	Development of consciousness	15.96 (4.08)	
Knowledge and experience in pain control (0–150)	Knowledge	40.91 (12.47)	81.44 (18.30)
	Experience	40.45 (10.28)	

### 3.2 Correlation between variables and hierarchical multiple linear regression

The findings of Pearson and Spearman correlation tests revealed a significant relationship between emotional intelligence and age (*r* = 0.20, *p* = .003), academic degree (*r* = 0.15, *p* = .032), and duration of care (*r* = 0.15, *p* = .032). Also, the Pearson correlation test revealed a significant relationship between emotional intelligence and spiritual intelligence (*r* = 0.30, *p* < .001) (Table 3).

**TABLE 3:** The correlation matrix among main variables in family caregivers of cancer patients.

Variable	1 *r* (*p*‐value)	2 *r* (*p*‐value)	3 *r* (*p*‐value)	4 *r* (*p*‐value)
Emotional intelligence	1			
Coping	0.07 (0.316)	1		
Spiritual intelligence	**0.30 (<0.001)**	0.07 (0.261)	1	
Knowledge and experience in pain control	0.09 (0.174)	0.01 (0.803)	0.15 (0.100)	1

*Note*: Pearson correlation test. Statistically significant values are represented by bolded.

In the regression model, only the demographic variables that, based on the correlation test, had a significant relationship with emotional intelligence were included. There was no auto‐correlation between the residuals, according to the Durbin‐Watson value of 1.73. Between the predictor variables, there was no multicollinearity (Variance inflation factor >1). The initial stage of the study reveals that age, academic degree, and duration of care significantly influenced the regression model and accounted for 6.6% of the variation in emotional intelligence (*F* = 4.81, *p* = .003). In the subsequent stage, when spiritual intelligence was included in the regression model, all predictor factors accounted for 12.4% of the variations in emotional intelligence (*F* = 7.04, *p* < .001). The results demonstrated that the variables of age (*β* = 0.16, *p* = .021) and spiritual intelligence (*β* = 0.26, *p* < .001) could predict emotional intelligence (Table 4).

**TABLE 4:** Predictors of emotional intelligence in family caregivers of cancer patients.

Dependent variable	Independent variables	*B*	SE	*β*	*t*	*p* Value^a^	*R* ^2^
Emotional intelligence	Age	2.09	0.90	0.16	2.33	**0.021**	0.124
	Academic degree	0.76	0.90	0.06	0.84	0.398	
	Duration of care	1.23	0.88	0.10	1.39	0.165	
	Spiritual intelligence	0.15	0.03	0.26	3.86	**<0.001**	

Abbreviations: SE, standard error; β, unstandardized coefficients. Statistically significant values are represented by bolded.

^a^ Multiple regression analyses.

## 4 DISCUSSION

In Iranian family caregivers of cancer patients, age and spiritual intelligence could predict emotional intelligence, and the most significant impact was on spiritual intelligence. The positive correlation with age suggests that emotional intelligence may develop and improve over time, potentially influencing caregiving experiences and coping mechanisms. The association with academic degrees implies that higher education levels might contribute to the development of emotional intelligence among caregivers, potentially facilitating better emotional regulation and support provision. Moreover, the positive correlations between duration of care and spiritual intelligence highlight the potential role of experience and spirituality in shaping emotional intelligence among caregivers. In other regression models, personality traits,^33^ gendered self‐concepts,^34^ being young, female, having higher employment, and education levels^25^ are known as predictors of emotional intelligence.

The current study showed that the emotional intelligence of the participants was high. Qan'ir et al.'s systematic review (2022) also showed good emotional intelligence for family caregivers of cancer patients.^35^ The family member who takes care of the patient, among all the members, has a higher emotional intelligence. This family member has better understood the feelings and emotions of the sick person and has assumed the duty of care as the primary caregiver.

Age and emotional intelligence were positively and significantly correlated in our study, and age provided a predictor of emotional intelligence among family caregivers of cancer patients. Also, Baudelaire et al. identified a positive relationship between age and emotional intelligence in their study.^36^ Their age significantly influences the emotional maturity of a person. As we go through life and have various experiences, our emotional intelligence develops, and as a result, our competence increases.^36^ Consequently, life events play a cumulative role in the development of emotional intelligence.

According to the results, a significant relationship was seen between academic degree and emotional intelligence in family caregivers of cancer patients. A meta‐analysis in 2020 showed that emotional intelligence was a significant predictor of learning ability and was highly associated with learning. Placing individuals in academic environments improves their social capabilities through formal and informal training.^37^ According to this evidence, the structure of emotional intelligence will improve under the influence of the environment and education. Therefore, it is suggested that ways to increase moral intelligence should be taught to family caregivers of cancer patients. In addition, encouraging the family member with higher education to accept the role of the caregiver could be a helping component of the healing process.

Also, the results showed that the emotional intelligence of the caregivers is higher in the more extended care period. This result indicates that the caregiver's understanding of the patient increases. It is also possible that these caregivers were more empathic, compassionate, caring, and resilient^36^ and stayed with the patient for extended care.

We revealed that the spiritual intelligence of our participants was at a reasonable level. Higher emotional intelligence brings deeper insight, courage, patience, and wisdom when faced with the hardships of life. Qan'ir et al. also found that several patients and their caregivers tended to spiritual activities.^35^ There was a statistically significant relationship between spiritual and emotional intelligence, a predictive factor for emotional intelligence among family caregivers of cancer patients. In line with our findings, Sogolitappeh et al., in their research, reported a positive and significant relationship between emotional and spiritual intelligence.^38^ Moreover, Beauvais et al. (2014) illustrate in a study that a significant correlation exists between emotional intelligence and spirituality. Emotional intelligence can develop spiritual growth, learning, and well‐being.^39^ Since emotional and spiritual are considered aspects of intelligence, these two concepts' positive coalition is evident.

Our study showed that the mean score of the total coping of family caregivers of cancer patients was high, and the caregivers mainly used approach coping strategies. A similar study conducted in Vietnam showed that family caregivers of individuals who have cancer also used approach coping strategies.^15^ A study from South Africa also suggests the same findings.^1^ People who use approach coping strategies experience less anxiety, depression, hopelessness, and, in retrospect, a higher quality of life. Increasing the social support of family caregivers facing cancer patients can cause coping success.^15^ One of the possible reasons behind the use of approach coping among family caregivers could be their higher emotional intelligence.

Our findings showed a positive correlation between emotional intelligence and coping, but this correlation needed to be proven significant. The study by Gomez et al. showed that emotional intelligence influences the coping approach of family caregivers of patients inflicted with Dementia. They stated that coping and higher emotional intelligence could aid caregivers with better management of their emotional responses.^40^

Our study showed that the participants had a high mean score of knowledge and experience about pain. The studies of Ma et al. showed that family caregivers had a much worse cancer pain experience^23^ and lesser knowledge of it^21^ than their patients. The level of knowledge and experience about pain among family caregivers of cancer patients is one of the results of pain management.^23^ Educational intervention in promoting family caregivers and their patients can lead to a better understanding of the emotional state of the patient and family, improve pain management results, and improve the quality of life of cancer patients and their families.

Based on this study, there is a positive correlation between emotional intelligence and knowledge and experience of pain, but this correlation was not statistically significant. Issa et al. found a vital relationship between emotional intelligence and the pain management of cancer patients. They state that emotional intelligence can be considered one of the main factors in pain management.^41^ Therefore, further research is needed to understand better the correlation between emotional intelligence and understanding pain and pain management experience.

One of the strengths of this study is its ability to investigate and measure the correlation between multiple concepts and emotional intelligence. Another strength is the large sample size and high response rate, which reached 94%.

### 4.1 Clinical implications

The identified relationships in the results of this study have important practical implications for support programs and interventions. By recognizing the factors associated with emotional intelligence among family caregivers, healthcare professionals can design targeted interventions to enhance emotional intelligence skills and provide tailored support. Interventions could focus on promoting emotional regulation strategies, facilitating education and training opportunities for caregivers, and integrating spiritual care into supportive interventions. In summary, the significant relationships between emotional intelligence and age, academic degree, duration of care, and spiritual intelligence contribute to understanding emotional intelligence among family caregivers of cancer patients. These findings underscore the importance of considering these factors in designing effective support programs and interventions for caregivers.

Healthcare providers can leverage the strengths of family caregivers of cancer patients, such as their good emotional intelligence, spiritual intelligence, coping skills, knowledge, and pain control experience, to provide better care for cancer patients. By working collaboratively with family caregivers, healthcare providers can utilize these strengths to improve care outcomes for cancer patients. Additionally, the study recommends using older family caregivers to care for cancer patients. Therefore, healthcare providers can consider the age of family caregivers when assigning caregiving roles to ensure that patients receive the best possible care.

### 4.2 Study limitations

This study has several limitations. First, using multiple questionnaires resulted in fewer willing participants, and the sampling process took longer than anticipated. To mitigate this issue, we recommended that participants be given more time to complete the questionnaires at their convenience and submit them at the subsequent meeting. Second, relying on self‐reported data collection tools may have introduced response bias and social desirability effects. Participants may have been inclined to provide answers they deemed socially acceptable or aligned with their desired image. Another limitation of this study is the lack of clear criteria specifying the cancer stage in the inclusion criteria for patients. Obtaining accurate and comprehensive information regarding the cancer stage proved challenging. We employed the duration of the family's involvement in the disease process as an alternative criterion.

Additionally, the study was constrained by the absence of data on the duration and frequency of analgesic administration. This information could have provided valuable insights into the potential impact on patient outcomes. Furthermore, while practical, convenience sampling may introduce limitations, such as the possibility of selection bias. Consequently, the generalizability of the findings may be affected. In conclusion, it is important to acknowledge these limitations, which may impact the interpretation and generalizability of the study's findings. Future research should address these limitations and employ more comprehensive methodologies to enhance the validity and reliability of the results.

## 5 CONCLUSIONS

The results of this study demonstrated in Iranian family caregivers of cancer patients that emotional intelligence, spiritual intelligence, coping, knowledge, and pain control experience were good. The findings showed a significant relationship between emotional intelligence and age, academic degree, duration of care, and spiritual intelligence. Age and spiritual intelligence could predict emotional intelligence; the most significant impact was on spiritual intelligence. This study recommends using older family caregivers to care for cancer patients and developing and implementing a training program to foster spiritual intelligence to improve emotional intelligence in family caregivers.

## AUTHOR CONTRIBUTIONS

**Shakiba Moosivand:** Data curation (equal); investigation (equal); methodology (equal); resources (equal); writing – original draft (equal); writing – review and editing (equal). **Omid Nazari:** Conceptualization (equal); data curation (lead); investigation (equal); methodology (equal); writing – original draft (equal); writing – review and editing (equal). **Ali Shahverdi:** Conceptualization (equal); data curation (equal); investigation (equal); methodology (equal); writing – original draft (equal); writing – review and editing (equal). **Mohammad Gholami:** Conceptualization (lead); investigation (equal); methodology (equal); supervision (supporting); validation (supporting); writing – original draft (supporting); writing – review and editing (equal). **Rasool Mohammadi:** Conceptualization (equal); formal analysis (lead); investigation (equal); methodology (equal); software (lead); validation (equal); writing – original draft (equal); writing – review and editing (equal). **Sajad Yarahmadi:** Conceptualization (equal); data curation (supporting); formal analysis (supporting); investigation (equal); methodology (equal); project administration (lead); resources (equal); software (supporting); supervision (lead); validation (lead); writing – original draft (lead); writing – review and editing (lead).

## FUNDING INFORMATION

No funds, grants, or other support were received.

## CONFLICT OF INTEREST STATEMENT

There is no conflict of interest between the authors.

## ETHICS STATEMENT

According to the guidelines of the Helsinki Declaration, this study was conducted. The Lorestan University of Medical Sciences Ethics Committee in Khorramabad, Iran, approved it (Code: I.R.LUMS.REC.1399.107). The participants provided their written, informed consent.

## Data Availability

The datasets generated during and analyzed during the current study are available from the corresponding author upon reasonable request.

## References

[1] Akpan‐Idiok PA , Ehiemere IO , Asuquo EF , Chabo JAU , Osuchukwu EC . Assessment of burden and coping strategies among caregivers of cancer patients in sub‐Saharan Africa. World. J Clin Oncol. 2020;11(12):1045‐1063. doi:10.5306/wjco.v11.i12.1045 PMC7769710 33437666

[2] Kwekkeboom KL . Cancer symptom cluster management. Semin Oncol Nurs. 2016;32(4):373‐382. doi:10.1016/j.soncn.2016.08.004 27789073 PMC5143160

[3] Kristanti MS , Effendy C , Utarini A , Vernooij‐Dassen M , Engels Y . The experience of family caregivers of patients with cancer in an Asian country: a grounded theory approach. Palliat Med. 2019;33(6):676‐684. doi:10.1177/0269216319833260 30916614 PMC6537031

[4] Kilic ST , Oz F . Family Caregivers' involvement in caring with cancer and their quality of life. Asian Pac J Cancer Prev. 2019;20(6):1735‐1741. doi:10.31557/apjcp.2019.20.6.1735 31244294 PMC7021632

[5] Kim Y , Carver CS , Cannady RS . Caregiving motivation predicts Long‐term spirituality and quality of life of the caregivers. Ann Behav Med. 2015;49(4):500‐509. doi:10.1007/s12160-014-9674-z 25637107

[6] Johansen S , Cvancarova M , Ruland C . The effect of cancer Patients' and their family Caregivers' physical and emotional symptoms on caregiver burden. Cancer Nurs. 2018;41(2):91‐99. doi:10.1097/ncc.0000000000000493 28426539

[7] Kaminska AO , Pshuk NG , Martynova YY . Social and emotional intelligence as a basis for communicative resource formation in family caregivers of patients with endogenous mental disorders. Wiad Lek. 2020;73(1):107‐112. doi:10.36740/WLek202001121 32124818

[8] Mirzaei S , Tame AI , Anbiaie R , Moradipour F , Nasiri M , Rohani C . Emotional intelligence as a predictor of health‐related quality of life in breast cancer survivors. Asia Pac J Oncol Nurs. 2019;6(3):261‐268. doi:10.4103/apjon.apjon_76_18 31259222 PMC6518981

[9] Vigna PM , de Castro I , Fumis RRL . Spirituality alleviates the burden on family members caring for patients receiving palliative care exclusively. BMC Palliat Care. 2020;19(1):77. doi:10.1186/s12904-020-00585-2 32493301 PMC7271458

[10] Vespa A , Spatuzzi R , Merico F , et al. Spiritual well‐being associated with personality traits and quality of life in family caregivers of cancer patients. Support Care Cancer. 2018;26(8):2633‐2640. doi:10.1007/s00520-018-4107-3 29460194

[11] La IS et al. Spirituality among family caregivers of cancer patients: the spiritual perspective scale. Res Nurs Health. 2020;43(4):407‐418. doi:10.1002/nur.22044 32515862 PMC7805015

[12] Jamalnia S , Javanmardifard S , Ghodsbin F , Zarea K , Ezzati E . The relationship between spiritual intelligence and emotional intelligence in patients with type 2. Diabetes. 2018;7(3):e79182. doi:10.5812/jjcdc.79182

[13] Dev RDO , Kamalden TFT , Geok SK , Abdullah MC , Ayub AFM , Ismail IA . Emotional intelligence, spiritual intelligence, SelfEfficacy, and health behaviors: implications for quality health. Int J Acad Res Busin Social Sci. 2018;8(7):794‐809. doi:10.6007/IJARBSS/v8-i7/4420

[14] Safavi M , Yahyavi ST , Narab HF , Yahyavi SH . Association between spiritual intelligence and stress, anxiety, and depression coping styles in patients with cancer receiving chemotherapy in university hospitals of Tehran university of medical science. J Cancer Res Ther. 2019;15(5):1124‐1130. doi:10.4103/jcrt.JCRT_382_17 31603122

[15] Long NX et al. Coping strategies and social support among caregivers of patients with cancer: a cross‐sectional study in Vietnam. AIMS. Public Health. 2021;8(1):1‐14. doi:10.3934/publichealth.2021001 PMC7870390 33575403

[16] Ghafoor H , Ahmad RA , Nordbeck P , Ritter O , Pauli P , Schulz SM . A cross‐cultural comparison of the roles of emotional intelligence, metacognition, and negative coping for health‐related quality of life in German versus Pakistani patients with chronic heart failure. Br J Health Psychol. 2019;24(4):828‐846. doi:10.1111/bjhp.12381 31290198

[17] Ke T , Barlas J . Thinking about feeling: using trait emotional intelligence in understanding the associations between early maladaptive schemas and coping styles. Psychol Psychother. 2020;93(1):1‐20. doi:10.1111/papt.12202 30369013 PMC7028072

[18] Wei X , Yu H , Dai W , et al. Discrepancy in the perception of symptoms among patients and healthcare providers after lung cancer surgery. Support Care Cancer. 2022;30(2):1169‐1179. doi:10.1007/s00520-021-06506-0 34448942

[19] Tang L , Yu H , Dai W , et al. Symptom trajectories informing patient care after lung cancer surgery: a longitudinal patient‐reported outcome study. Ann Surg Oncol. 2023;30(5):2607‐2617. doi:10.1245/s10434-022-13065-z 36658248

[20] Wei X , Yu H , Dai W , et al. Patient‐reported outcomes of video‐assisted thoracoscopic surgery versus thoracotomy for locally advanced lung cancer: a longitudinal cohort study. Ann Surg Oncol. 2021;28:1‐14. 10.1245/s10434-021-09981-1 33880671

[21] Ma X , Yu W , Lu Y , Yang H , Li X , Kang D . Pain knowledge of patients and family caregivers as predictors of pain management outcomes in cancer patients: a multicenter study in China. Support Care Cancer. 2022;30(1):575‐584. doi:10.1007/s00520-021-06457-6 34347180

[22] Smyth JA , Dempster M , Warwick I , Wilkinson P , McCorry NK . A systematic review of the patient‐ and Carer‐related factors affecting the experience of pain for advanced cancer patients cared for at home. J Pain Symptom Manag. 2018;55(2):496‐507. doi:10.1016/j.jpainsymman.2017.08.012 28843458

[23] Ma X , Yu W , Lu Y , Yang H , Li X , Kang D . Congruence of cancer pain experience between patients and family caregivers and associated factors: a multicenter cross‐sectional study in China. Support Care Cancer. 2021;29(10):5983‐5990. doi:10.1007/s00520-021-06156-2 33770256

[24] Fariselli L , Ghini M , Freedman J . Age and emotional intelligence. The Emotional Intelligence Network Six Seconds; 2008:1‐10.

[25] Stami T , Ritin F , Dominique P . Demographic predictors of emotional intelligence among radiation therapists. J Med Radiat Sci. 2018;65(2):114‐122. doi:10.1002/jmrs.277 29687618 PMC5986065

[26] Hagi H , Nazemi A , Karimollahi M . Role of personality factors and religious beliefs in resiliency of patients with esophageal cancer. J Health Care. 2016;18(2):150‐160. http://hcjournal.arums.ac.ir/article-1-549-en.html

[27] Wong C‐S , Law KS . The effects of leader and follower emotional intelligence on performance and attitude: an exploratory study. Leadersh Q. 2002;13(3):243‐274. doi:10.1016/S1048-9843(02)00099-1

[28] Sharif Nia H et al. Psychometric properties of the king spiritual intelligence questionnaire (KSIQ) in physical veterans of Iran‐Iraq warfare. J Milit Med. 2015;17(3):145‐153. http://eprints.goums.ac.ir/id/eprint/4668

[29] Nanthini B , Rathnasabapathy M , Angeline J . Perceived‐stress and coping strategies among school and college students during COVID‐19 lockdown: a cross sectional‐survey from Tamil Nadu. Int J Indian Psychol. 2021;9(4):2249‐2256. doi:10.25215/0904.211

[30] Ferrell BR , Rhiner M , Rivera LM . Family Pain Questionnaire (FPQ) 2012. https://www.midss.org/content/family-pain-questionnaire-fpq

[31] Lawshe CH . A quantitative approach to content validity. Pers Psychol. 1975;28(4):563‐575.

[32] Polit DF , Beck CT , Owen SV . Is the CVI an acceptable indicator of content validity? Appraisal and recommendations. Res Nurs Health. 2007;30(4):459‐467. doi:10.1002/nur.20199 17654487

[33] Alghamdi NG , Aslam M , Khan K . Personality traits as predictor of emotional intelligence among the university teachers as advisors. Educ Res Int. 2017;6:9282565. doi:10.1155/2017/9282565

[34] Martínez‐Marín MD , Martínez C , Paterna C . Gendered self‐concept and gender as predictors of emotional intelligence: a comparison through of age. Curr Psychol. 2021;40(9):4205‐4218. doi:10.1007/s12144-020-00904-z

[35] Qan'ir Y et al. Quality of life among patients with cancer and their family caregivers in the sub‐Saharan region: a systematic review of quantitative studies. PLOS. Glob Public Health. 2022;2(3):e0000098. doi:10.1371/journal.pgph.0000098 PMC10021310 36962119

[36] Budler LC et al. Emotional Intelligence among Nursing Students: Findings from a Longitudinal Study. Emotional Intelligence among Nursing Students: Findings from a Longitudinal Study. In Healthcare. MDPI; 2022. doi:10.3390/healthcare10102032 PMC9601576 36292477

[37] Vázquez FL , Otero P , Díaz O , Sánchez T , Pomar C . Emotional intelligence in women caregivers with depressive symptoms. Psychol Rep. 2011;108(2):369‐374. doi:10.2466/09.13.14.Pr0.108.2.369-374 21675552

[38] Sogolitappeh FN , Hedayat A , Arjmand MR , Khaledian M . Investigate the relationship between spiritual intelligence and emotional intelligence with resilience in undergraduate (BA) students. Int Lett Soc Human Sci. 2018;82(4):10‐18. doi:10.18052/www.scipress.com/ILSHS.82.10

[39] Beauvais A , Stewart JG , DeNisco S . Emotional intelligence, and spiritual well‐being: implications for spiritual care. J Christ Nurs. 2014;31(3):166‐171. doi:10.1097/cnj.0000000000000074 25004728

[40] Gómez‐Trinidad MN , Chimpén‐López CA , Rodríguez‐Santos L , Moral MA , Rodríguez‐Mansilla J . Resilience, emotional intelligence, and occupational performance in family members who are the caretakers of patients with dementia in Spain: a cross‐sectional, analytical, and descriptive study. J Clin Med. 2021;10(18):4262. doi:10.3390/jcm10184262 34575373 PMC8469665

[41] Issa MR , Muslim NA , Alzoubi RH , et al. The relationship between emotional intelligence and pain management awareness among nurses. Healthcare (Basel). 2022;10(6):1047. doi:10.3390/healthcare10061047 35742097 PMC9222258

